# Hypertension and Arrhythmias: A Clinical Overview of the Pathophysiology-Driven Management of Cardiac Arrhythmias in Hypertensive Patients

**DOI:** 10.3390/jcdd9040110

**Published:** 2022-04-06

**Authors:** Jacopo Marazzato, Federico Blasi, Michele Golino, Paolo Verdecchia, Fabio Angeli, Roberto De Ponti

**Affiliations:** 1 Department of Medicine and Surgery, University of Insubria, 21100 Varese, Italy; j.marazzato88@gmail.com (J.M.); federico.blasi.md@gmail.com (F.B.); micheleg1390@gmail.com (M.G.); angeli.internet@gmail.com (F.A.); 2 Fondazione Umbra Cuore e Ipertensione-ONLUS, 06100 Perugia, Italy; verdecchiapaolo@gmail.com; 3 Division of Cardiology, Hospital S. Maria della Misericordia, 06100 Perugia, Italy; 4 Department of Medicine and Cardiopulmonary Rehabilitation, Maugeri Care and Research Institute, IRCCS Tradate, 21049 Tradate, Italy

**Keywords:** hypertension, atrial fibrillation, primary hyperaldosteronism, antihypertensive agents, artificial pacemakers, anticoagulants

## Abstract

Because of demographic aging, the prevalence of arterial hypertension (HTN) and cardiac arrhythmias, namely atrial fibrillation (AF), is progressively increasing. Not only are these clinical entities strongly connected, but, acting with a synergistic effect, their association may cause a worse clinical outcome in patients already at risk of ischemic and/or haemorrhagic stroke and, consequently, disability and death. Despite the well-known association between HTN and AF, several pathogenetic mechanisms underlying the higher risk of AF in hypertensive patients are still incompletely known. Although several trials reported the overall clinical benefit of renin–angiotensin–aldosterone inhibitors in reducing incident AF in HTN, the role of this class of drugs is greatly reduced when AF diagnosis is already established, thus hinting at the urgent need for primary prevention measures to reduce AF occurrence in these patients. Through a thorough review of the available literature in the field, we investigated the basic mechanisms through which HTN is believed to promote AF, summarising the evidence supporting a pathophysiology-driven approach to prevent this arrhythmia in hypertensive patients, including those suffering from primary aldosteronism, a non-negligible and under-recognised cause of secondary HTN. Finally, in the hazy scenario of AF screening in hypertensive patients, we reviewed which patients should be screened, by which modality, and who should be offered oral anticoagulation for stroke prevention.

## 1. Introduction

The overall prevalence of hypertension (HTN) in adults is roughly 30–45% [1] and becomes even more common with advancing age [2]. HTN is also a well-known risk factor for atrial fibrillation (AF) [3,4,5], which may even occur when borderline values of blood pressure (BP) are recorded [6,7,8,9,10]. Moreover, AF exerts an important prognostic role in hypertensive patients, thus potentially leading to ischaemic and haemorrhagic stroke, hospitalisations for heart failure, and, in the worst circumstances, death [10,11,12]. Therefore, it stands to reason that primary prevention measures devoted to reducing incident AF are required to avoid potentially troublesome cardiac and cerebrovascular events which may occur in this clinical scenario. Moreover, increasing age and the associated burden of other comorbidities such as diabetes mellitus, heart failure, coronary artery disease, chronic kidney disease, obesity, and obstructive sleep apnea would synergistically act with HTN as major contributors to AF development and progression [10].

Through a review of the available literature, we investigated the pathophysiological mechanisms responsible for incident AF in hypertensive patients. The aim of this review was therefore to summarise a pathophysiology-driven, patient-tailored approach to prevent the onset of cardiac arrhythmias, namely atrial fibrillation, in the general population affected by HTN. To underscore the importance of a pathophysiological approach to HTN, a dedicated focus has also been reported on which hypertensive patients would greatly benefit from specific treatment options in the setting of primary hyperaldosteronism, a non-negligible cause of secondary HTN. 

Moreover, when AF nonetheless develops as an unavoidable consequence of atrial myopathy, it should be recognised in a timely manner to avoid potentially harmful consequences. Although several issues do exist about the possibility of AF screening in hypertensive patients, in this hazy scenario, we investigated the available modalities to detect silent/subclinical AF episodes in hypertensive patients and which patients should be offered oral anticoagulation for stroke prevention.

## 2. Materials and Methods

We performed a bibliographic research on Medline considering manuscripts published up to 2021, according to the following Boolean research strings: “Arterial hypertension AND arrhythmias”, “Arterial hypertension AND atrial fibrillation”, “Arterial hypertension AND supraventricular arrhythmias”. The literature research was independently conducted by two authors (FB and JM) and then revised by JM, FB, and MG, who reached a shared decision by consensus in case of discordance.

## 3. Common Pathophysiological Aspects Explaining the Link between Hypertension and Cardiac Arrhythmias

As observed in animal models, HTN per se is associated with ion channel imbalance and the progressive development of myocardial fibrosis in hypertensive hearts [13,14,15]. The ensuing molecular and structural alterations would therefore represent a fertile substrate for arrhythmogenesis. On the one hand, HTN-related shear stress would lead to both a long outward potassium (K^+^) current (Kv1.5) [13] and the altered release of intracellular calcium (Ca^2+^) from the sarcoplasmic reticulum [14], thus leading to a shorter action potential duration and delayed afterdepolarizations (DAD) in myocardial cells, respectively. In fact, a shorter action potential duration would predispose to enhanced automatism and re-entrant mechanisms [16]. In addition to ion channel abnormalities, HTN is also associated with maladaptive gap junction remodelling due to the abnormal expression of gap junction proteins such as connexin 43 and 40 [15,17] which would determine the abnormal conduction properties and fibrotic evolution of myocardial tissue, thus prompting nonuniform anisotropy, slow conduction, and, therefore, arrhythmogenesis in hypertensive hearts. In addition to this, cardiovascular risk factors, HTN included, are accompanied by low-grade inflammation and oxidative stress, which further promote ion channels and connexin downregulation/dysfunction, abnormal Ca^2+^ handling, and, finally, the activation of profibrotic signaling, which would all promote arrhythmogenesis [18].

**Figure 1:**
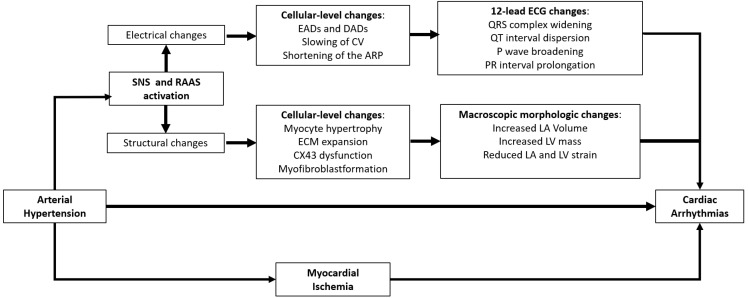
Electro-pathological and clinical changes occurring in hypertensive hearts. ARP, atrial refractory period; SNS, sympathetic nervous system; CV, conduction velocity; CX43, connexin 43; DADs, delayed afterdepolarizations; EADs, early afterdepolarizations; ECM, extracellular matrix; LA, left atrial; LA Vol, left atrial volume; LV, left ventricular; RAAS, renin–angiotensin–aldosterone system. See text for further details.

Furthermore, as displayed on Figure 1, the HTN-related activation of the renin–angiotensin–aldosterone (RAA) cascade and sympathetic nervous system (SNS), in addition to myocardial ischemia in hypertrophic hearts, would also play a major role in the pathogenesis of cardiac arrhythmias in HTN [19,20]. All these mechanisms are discussed in the next sections of this article.

### 3.1. Myocardial Electro-Pathological Remodelling in Arterial Hypertension: The Key Role of Renin–Angiotensin–Aldosterone and Sympathetic Nervous Systems

Different hormone systems are involved in this complex scenario. On the one hand, angiotensin II, a mediator of RAAS, does modulate specific ion currents in cardiac myocytes, including L- and T-type inward Ca^2+^ [21,22] and K^+^ currents [23]. Moreover, in a murine model, aldosterone seems to increase the molecular expression of L-type Ca^2+^ channels while reducing the activity of both delayed rectifier (IKr) and transient outward K^+^ currents (Ito1) [24]. Aldosterone has also been shown to induce Ca^2+^ overload due to the opening of ryanodine receptors in the sarcoplasmic reticulum [25], which may increase delayed afterdepolarizations (DAD), thereby raising the chance of cardiac arrhythmias mediated by triggered activity [26]. In this regard, Pluteanu et al. [27] demonstrated the existence of subcellular alterations in Ca^2+^ handling in spontaneous hypertensive rats, which were associated with an increased propensity of atrial myocytes to develop frequency-dependent and arrhythmogenic Ca^2+^ alternans, a mechanism potentially triggering cardiac arrhythmias. 

In addition to ion channel modifications, the RAAS plays a key role in the progression of atrial and ventricular fibrosis through the proliferation of fibroblasts in the extracellular matrix [19,20]. In fact, myocardial fibrosis, associated with connexin dysregulation, generally leads to slow and heterogeneous conduction velocity, nonhomogenous impulse propagation, and re-entrant atrial and ventricular arrhythmias [11,28]. HTN, as well as the ensuing LV hypertrophy, may also cause an abnormal expression of junctional complexes, which have been associated with greater myocardium vulnerability [15,29]. Moreover, the imbalance between oxygen demand and supply occurring in this setting would further activate myofibroblasts and induce hypertrophic modifications in vascular smooth muscle cells [29,30], thus leading to a vicious cycle made up of collagen deposition [31], progressive myocyte hypertrophy, and diastolic dysfunction [32], which is regarded as the first compensatory pathophysiological response in hypertensive hearts [33]. In addition to these mechanisms, SNS would also lead to enhanced RAAS activity and HTN-induced LV afterload, with a remarkable synergistic effect on arrhythmia onset in hypertrophic hearts [20].

From a pathophysiological perspective, diastolic dysfunction generally causes a reduction in LA passive emptying, thus increasing LA pressures during atrial diastole and eventually causing LA enlargement [34]. Over time, the progressive distension and stretching of the LA and pulmonary veins may induce an electrical remodelling of these anatomical chambers, thus leading to shorter atrial effective refractory periods [35], the greater dispersion of atrial repolarisation and, therefore, vulnerability to AF [36,37]. LA stretching would also prompt electrical dissociation among muscle bundles, which would further facilitate the initiation and maintenance of multiple small re-entrant wavelets to sustain this cardiac arrhythmia [20]. 

As to the clinical implication of these pathophysiological mechanisms, AF episodes in hypertensive patients are greatly associated with the severity of LV myocardial stiffness or, in other words, the extent of diastolic dysfunction [38]. In this regard, as assessed on a vast patient cohort undergoing echocardiographic evaluation [38], Tsang et al. showed how the greater the degree of diastolic dysfunction, the higher the probability of AF episodes occurring [38]. Therefore, as shown on Figure 1, the increased LV mass, LV myocardial stiffness, and ensuing diastolic dysfunction and LA enlargement would all play a great role in the genesis of cardiac arrhythmias, namely AF, in hypertensive patients [39,40]. 

### 3.2. The Role of Myocardial Ischemia

Myocardial ischemia may lead to arrhythmogenesis in HTN due to mechanisms inherently connected to LVH or atherosclerotic disease involving the major epicardial coronary arteries. On the one hand, changes in arteriolar wall thickening and relative capillary density may lead to reduced microvascular flow in hypertrophic hearts [41,42,43]. However, HTN-mediated ischemia is not limited to small vessels only, and the global involvement of the coronary artery tree in hypertensive hearts well explains the overall risk of myocardial ischemia and scar formation in these patients [29]. In this regard, a strong connection between the obstruction of atrial coronary branches and AF occurrence in the setting of acute myocardial infarction has been described [44,45]. As observed in studies conducted on animal models [46], atrial ischemia and the ensuing LA stretching synergistically interact in leading to a reduced myocardial conduction velocity and an increased conduction heterogeneity, which would elicit myocardial vulnerability and AF. Of note, not only could atrial ischemia be the result of atherosclerotic heart disease, but pulmonary hypertension and the ensuing combination of hypoxia with increased atrial pressure may prompt AF by means of ischemic mechanisms [47]. 

Moreover, Kolvekar et al. described an association between atrial ischemia and the sclerosis of sinus node and atrioventricular node branches [48], and, as pointed out in a retrospective study conducted by Ciulla et al. [49], the prevalence of AF seems higher in patients with a diseased sinus node artery (41.2% vs. 7.4% *p* < 0.001). Hence, the ischemic damage caused by flow abnormality in the sinus node artery may undermine the structural integrity of the sinus node itself, thus determining a widespread structural and electrical atrial remodelling, which represents the underlying substrate to AF development in this clinical setting. 

However, not only AF could be the result of ischemic mechanisms involving atrial branches of epicardial coronary arteries (i.e., primary atrial ischemia), but ventricular ischemia could also be responsible for this cardiac arrhythmia (i.e., ventricular-induced or secondary atrial ischemia). On the one hand, atrial stretching occurring in the setting of myocardial infarction would increase the LA surface area, thus prolonging electrical conduction and facilitating AF initiation and maintenance [50]. However, the greater the ischemic involvement of the LV, the higher the incidence of AF. In a subanalysis of the CULPRIT-SHOCK (Culprit Lesion Only PCI versus Multivessel PCI in Cardiogenic Shock) trial, compared with patients with LV myocardial infarction and no cardiogenic shock (CS), the authors observed a significant incidence of AF in patients with CS: global ischemia induced by extensive LV involvement in CS, the ensuing extensive myocardial injury, and the increased LA size and pressure would all explain the remarkable prevalence of AF observed in this high-risk population [51]. Moreover, myocardial ischemia may also lead to the transmural dispersion of ventricular repolarization, which may favour early after depolarizations (EAD) and polymorphic ventricular tachycardias in the affected patients [20,21,29,52,53]. 

## 4. Arterial Hypertension and Atrial Fibrillation: Pathophysiology-Based Strategies to Prevent a Hazardous Association

Given the remarkable prevalence of AF in hypertensive patients, the clinical impact of blood pressure in relation to the occurrence of AF deserves special analysis. A pathophysiology-based approach to HTN in AF patients and a proposed algorithm for the early detection of AF in HTN are provided in the next sections of this article.

### 4.1. Clinical Implications of High Blood Pressure in Patients with Atrial Fibrillation

The presence of uncontrolled HTN in AF patients promotes the already described electro-anatomical atrial remodelling, which is responsible for AF evolution from paroxysmal to more persistent clinical forms of arrhythmia with an overall dismal prognosis in this patient population [54,55,56,57,58,59,60,61]. Indeed, in a large Swedish registry of AF patients on oral anticoagulants, Friberg et al. found that HTN was not only an independent predictor for thromboembolic complications but also of intracranial [HR 1.32, 95% CI (1.15–1.52)] and major bleedings [HR 1.25, 95% CI (1.16–1.33)] [62]. These results are indeed reflected by the integration of HTN in both CHA2DS2-VASc and HAS-BLED scores to estimate the thromboembolic and haemorrhagic hazard, as recommended by current guidelines on AF management [63,64]. However, it is still debated which, between a long-standing history of increased blood pressure and high systolic blood pressure values per se, portends a greater risk of ischemic and haemorrhagic events in hypertensive patients. In a vast community-based prospective registry, Ishii et al. showed that, in AF patients, only systolic blood pressure values beyond 150 mmHg were significantly associated with a higher risk of ischemic [HR 1.74, 95% CI (1.08–2.72)] and bleeding events [HR 2.01, 95% CI (1.21–3.23)] as compared with adequately matched normotensive cases [65]. Similar results were provided by a subanalysis of the Japanese J-RHYTHM AF registry, including more than 7046 patients with nonvalvular AF [66], suggesting that every clinician should aim at an adequate blood pressure control to improve outcome in AF patients. 

Moreover, HTN seems responsible for cardioembolic stroke through mechanisms which would act independently from AF. Although the SPRINT (Systolic Blood Pressure Intervention Trial) reported an exceedingly high risk of thromboembolic events in patients with pre-existent and new-onset AF, despite adequate blood pressure control [67], on the other hand, a body of evidence suggests that HTN per se could also directly promote left atrial thrombosis. In this regard, Zabalgoitia et al. [68] demonstrated a lower flow velocity and a higher risk of thrombosis in the left atrial appendix in hypertensive patients regardless of AF, with results confirmed by a subanalysis of the SPAF-III (Stroke Prevention in Atrial Fibrillation III) trial [69]. In hypertensive patients, endocardial thrombogenesis seems promoted by oxidative stress [70,71,72,73], which is increased by RAAS activation and by the subsequent inflammation occurring in diseased atria and in the left atrial appendage [74,75,76]. 

Therefore, blood pressure control is paramount to minimize the risk of myocardial ischemia, stroke, and oral-anticoagulant-related bleedings in AF cases. Until more data are available, blood pressure values in AF patients on oral anticoagulants should be at least <130 mmHg and <80 mmHg for systolic and diastolic blood pressure values, respectively [1,9,77], and oral anticoagulants should be used with caution in patients with persistent uncontrolled hypertension [10]. Moreover, in case of any clinical suspicion of myocardial ischemia, the prompt assessment of the atherosclerotic involvement of epicardial coronary arteries is mandatory in these patients.

Therefore, it stands to reason that primary prevention measures to prevent AF occurrence and the early detection of this arrhythmia [68] are paramount in patients diagnosed with HTN to avoid the described life-threatening major cerebral and cardiovascular events. 

### 4.2. Primary Prevention of Atrial Fibrillation: A Pathophysiology-Based Approach in Patients with Essential Hypertension

Given the predominant role of RAAS in the pathogenesis of AF, ACE inhibitors (ACEi) and angiotensin receptor blockers (ARB) seem a reasonable first-line treatment option in hypertensive patients. Moreover, LVH has been shown to be partially reversible after treatment with RAAS blockers, with studies demonstrating improved electrical and structural parameters and reduced AF burden following treatment with these agents [78,79,80]. 

In the Losartan Intervention For Endpoint reduction in hypertension (LIFE) study, 9193 hypertensive patients were randomized to once-daily losartan- or atenolol-based antihypertensive therapy to detect outcome differences regarding the long-term occurrence of new-onset AF. Compared with Atenolol, Losartan was associated with significantly fewer AF episodes and a better overall outcome [RR 0.67, 95% CI 0.55–0.83] [81]. 

ACEi or ARB could have an even greater role in avoiding AF occurrence in patients with LVH and systolic heart failure [82,83,84]. Although this effect has been attributed to the antifibrotic and antiapoptotic effect of these drugs, this superiority over betablockers is nonetheless quite surprising. Therefore, despite the intrinsic antiarrhythmic effect of betablockers, both cardiac fibrosis and negative remodelling play a central role in AF onset in hypertensive patients. Even when tested versus calcium antagonists, RAAS blockers showed a lower risk of AF development in a similar patient population [85]. 

Although RAAS blockers showed a net superiority over beta-blockers for AF prevention in HTN patients, their combined use seems beneficial in hypertensive patients suffering from heart failure. In a meta-analysis including 11,952 patients, Nasr et al. reported that betablockers significantly reduced the incidence of AF onset in heart failure, provided that a background treatment with ACEi was warranted, and similar outcomes were observed for MRA in similar patients [86]. Another clear benefit of betablockers is the well-known protection against sudden cardiac death [53,87]. 

In light of this evidence, betablockers seem to further support the action of RAAS blockers in AF prevention in hypertensive patients; however, they should not be regarded as a first-line therapy for HTN unless there is a specific indication for their use, such as heart failure, angina symptoms, or established AF [1]. 

In conclusion, although RAAS inhibitors do not seem to prevent AF recurrence in patients with an already established diagnosis of this cardiac arrhythmia [88,89,90], ACEi and ARB should be first offered to patients with essential hypertension to prevent incident AF. Although betablockers and MRA should be generally used in addition to ACEi and ARB in specific settings, MRA are to be first considered in specific subsets, such as patients suffering from primary aldosteronism (PA), a non-negligible cause of secondary hypertension.

Finally, there is strong evidence from preclinical research and clinical studies that targeting inflammation and oxidative stress may provide a path to ameliorate cardiac arrhythmia burden. Indeed, cardioprotective SGLT2 inhibitors, statins, and omega-3 fatty acids exhibit potent antioxidative and anti-inflammatory properties. These agents most likely affect the proarrhythmia primary mechanisms, such as triggered activity as well as profibrotic signalling. However, the causal relationship is missing, and further studies are required to assess the real impact of these drugs on arrhythmogenesis in hypertensive patients [18].

### 4.3. Focus on Primary Aldosteronism: An Under-Recognized Cause of Secondary Hypertension Prompting a Targeted Medical and Surgical Treatment

PA, also known as primary hyperaldosteronism or Conn’s syndrome, refers to the excess production of aldosterone essentially caused by hyperplasia or tumors involving adrenal glands and resulting in high blood pressure in the affected patients [91]. It is the most common endocrine cause of secondary hypertension, with a prevalence spanning from 4.3–9.5% in hypertensive patients to 17–23% in those with resistant HTN [92]. Moreover, a body of evidence suggests that PA confers a greater risk of stroke, AF, and cardiovascular disease than similar patient cohorts with essential hypertension [93]. 

As to associated cardiac arrhythmias, AF is by far the most observed rhythm disorder (7.2% prevalence on average), with other cardiac arrhythmias occurring in up to 5.2% of cases [94,95]. 

PA could promote arrhythmogenesis through different mechanisms [96]. On the one hand, aldosterone hypersecretion induces inflammation by producing reactive oxygen species which activate proinflammatory transcription factors in macrophages [74,75,76], causing cardiac interstitial macrophage infiltration with subsequent fibrosis [97]. On the other hand, through resting membrane hyperpolarization, Na^+^-K^+^ ATPase inhibition, and the suppression of K^+^ channel conductance, aldosterone-mediated hypokalemia further explains the mechanisms of arrhythmogenesis occurring in PA patients [98,99,100]. Accordingly, a study from the German Conn’s Registry confirmed that AF was more commonly found in patients with the hypokalemic variant of PA than in those with normal values of serum potassium levels [95]. 

Therefore, PA has targeted medical treatment and potentially curative surgical solutions, which may ameliorate the associated cardiovascular risks as well as the rate of incident AF. 

**Figure 2:**
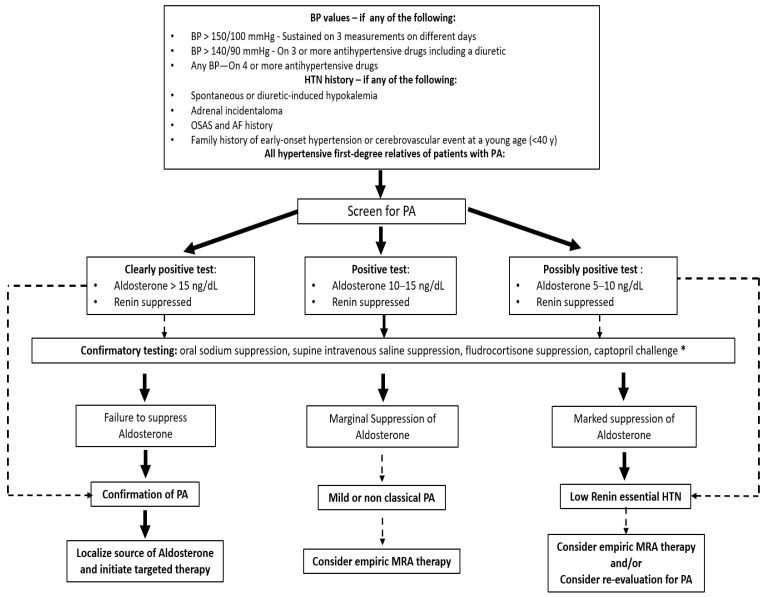
Screening and diagnostic approach for primary aldosteronism. A positive screen for primary aldosteronism should suggest high aldosterone levels and a suppressed renin activity. Confirmatory testing can be used in this setting. Solid arrows indicate recommended decision pathways; dashed arrows indicate other possible diagnostic alternatives in appropriate clinical contexts. * Confirmatory testing suggesting aldosterone hypersecretion: (1) oral sodium suppression (positive if 24 h urinary aldosterone excretion rate is greater than 12–14 mg/die); (2) supine intravenous saline suppression (positive if aldosterone levels are greater than 10 ng/dL after 2 L of saline infusion); (3) fludrocortisone suppression (positive if seated aldosterone greater than 6 ng/dL with plasma renin activity lower than 1.0 ng/mL/h); and, finally, (4) captopril challenge (positive if less than 30% suppression of aldosterone from baseline while plasma renin activity remains suppressed post 25 mg of oral captopril). AF, atrial fibrillation; BP, blood pressure; HTN, hypertension; MRA, mineralcorticoid-receptor antagonists; OSAS, obstructive sleep apnea syndrome; PA, primary aldosteronism. (Modified and adapted from document of The Endocrine Society [101]).

**Figure 3:**
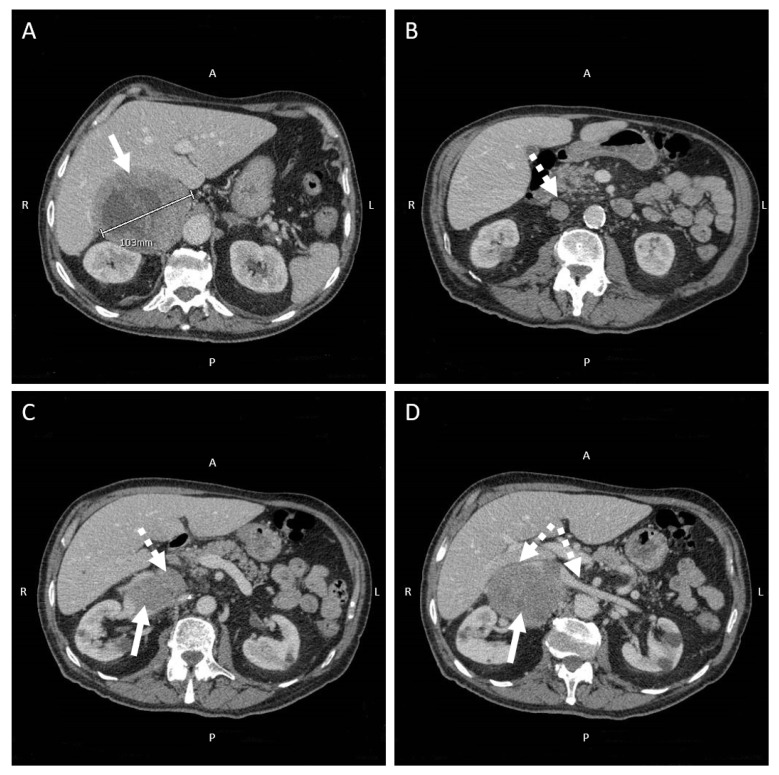
(**A**–**D**). Computed tomography of the abdomen in a patient with suspected primary aldosteronism. A 78-year-old patient referred to medical attention for dysregulated hypertension, irregular heartbeat, and remarkable peripheral oedema. Upon admission, 12-lead ECG showed atrial fibrillation with high ventricular rate. Transthoracic echocardiogram displayed signs of moderate left ventricular hypertrophy only. Laboratory tests showed remarkably low potassium levels together with high levels of serum aldosterone and suppressed renin activity. Therefore, computed tomography scan of the abdomen with iodine contrast administration was then carried out to identify any adrenal mass (**A**–**D**). A 10 cm, bulky adrenal tumor is well evident from cross-sectional imaging acquired during the arterial phase (**A**). The mass (white arrows) shows hypodense foci and colliquative areas with signs of compression of the neighboring anatomical structures. From a caudal to a more cranial perspective, the inferior vena cava and renal veins are progressively compressed and anteriorly dislodged by the adrenal mass (dashed arrows, **B**–**D**), thus explaining the remarkable peripheral oedema clinically observed in this patient. The patient is currently scheduled for abdominal video laparoscopy for adrenal mass excision and the ensuing histopathologic characterization.

Figure 2 shows a flowchart helping the clinician to diagnose and manage PA when clinically suspected [101]. Broadly speaking, patients who demonstrate a suppressed renin, markedly elevated aldosterone (i.e., when plasma aldosterone concentration is greater than 550 pmol/L or 15 ng/dL), and spontaneous hypokalemia can also be diagnosed with PA without confirmatory testing [102]. When PA diagnosis is confirmed, dietary sodium restriction and medical treatment with MRA should be immediately offered, not only to reduce blood pressure but also to lower the overall risk of AF occurrence [103]. However, surgical adrenalectomy seems associated with even better long-term cardiovascular outcomes [101], a further reduction in AF occurrence, and other major cardiovascular comorbidities in these patients [104]. Therefore, in the case of PA diagnosis, cross-sectional imaging is required to localise the adrenal mass, thus prompting further surgical evaluation in selected PA cases (Figure 3).

## 5. Early Detection of Atrial Fibrillation in Hypertensive Patients: A Proposed Algorithm

Despite all efforts to prevent AF in hypertensive patients, structural heart disease and atrial cardiomyopathy in this setting would nonetheless cause progressive atrial derangement and electrical vulnerability, thus promoting a vicious cycle known as “atrial failure”, which is intimately connected with AF development [105]. 

It is well known that AF is a potentially life-threatening cause of cerebral thromboembolism, and clinically silent forms might wreak even greater havoc if not recognised in a timely manner. For these reasons, hypertensive patients with an uncertain history of AF and evidence of prior cerebrovascular events should be accurately studied to differentiate strokes of cardioembolic origin from those secondary to atherosclerotic disease or cerebral haemorrhage [106]. In these cases, ECG monitoring can be helpful to identify patients with clinically silent AF [107,108], and, in the case of cryptogenic stroke, an implantable cardiac monitor (ICM) should be considered [10]. Over the last decade, cardiac implantable electronic devices (CIEDs) [109], ICM included [110], have proved extremely helpful in the early detection of subclinical AF episodes, but it is still debated which arrhythmic burden should prompt immediate oral anticoagulation in these patients. For the sake of clarity, clinically silent AF is defined for asymptomatic arrhythmia episodes detected on 12-lead ECG or an ECG strip; conversely, subclinical AF is represented by arrhythmia detected by CIEDs [10]. However, differentiating clinical from subclinical AF is not a matter of mere speculation. In fact, subclinical AF seems to portend a lower thromboembolic risk compared with clinical AF [111], and no clear cause–effect relationship between subclinical AF and ischemic stroke has been clearly proven in this setting [109]. However, the longer the duration of subclinical AF episodes, the greater their association with thromboembolic events [112]. For this reason, a recent European Heart Rhythm Association (EHRA) consensus document suggested oral anticoagulation administration for subclinical AF episodes longer than 5.5 h/day only when a significant risk of cerebral thromboembolism is established (i.e, CHA2DS2Vasc scores ≥ 2 and 3 in men and women, respectively) [111]. Whether this strategy pays off in terms of better clinical outcome is unclear. In fact, by randomising elderly patients with stroke risk factors and no AF history to the ICM strategy or usual care, the LOOP study did not prove the superiority of ICM over controls in terms of better clinical outcome after early AF detection [110]. Several issues raised by the same investigators might explain the overall negative results of this trial, such as the inadequate estimate of the primary outcome event rate, the relatively short duration of follow-up, and the initiation of oral anticoagulation for subclinical episodes lasting as low as 6 min. In keeping with prior observations [112], these results would suggest that not all subclinical AF episodes may benefit from early anticoagulation, and two ongoing randomized controlled trials might provide clearer answers in patients with CIEDs [113,114]. 

Moreover, in this already hazy scenario, it is all but crystal-clear which hypertensive patients with neither stroke history nor CIEDs/ICM should be screened for silent AF, and, not least, through which modality. On the one hand, the burden of cardiovascular comorbidities and blood biomarkers might play an important role in identifying people at a sufficient risk to warrant AF screening [115]. The thorough assessment of the *P* wave morphology on surface ECG may also be useful in identifying potential risk markers for AF, such as prolonged *P* wave duration, left atrial enlargement, and advanced interatrial (i.e., Bachmann bundle) block. [116]. Similar observation can be made for LVH, diastolic dysfunction, and left atrial enlargement as assessed on transthoracic echocardiogram [116]. However, what would be the best approach for AF screening in high-risk patients? On one side of the spectrum of the available modalities for AF screening, on account of the low cost and the great sensitivity yield, radial pulse taking should be regarded as the first option to be offered in patients aged ≥65 years and deemed at high risk of developing AF. Surface ECG analysis in the case of arrhythmic pulse is therefore warranted, and, if clinical AF is confirmed, oral anticoagulation should be promptly administered according to the patient’s thromboembolic risk profile [10]. Furthermore, a variety of screening technologies have been developed over the years and with progressively better AF detection accuracy [117], but no comparative trials have been carried out so far with any of these devices. Accordingly, European guidelines on AF diagnosis and management [10] strongly recommend a single-lead ECG tracing of ≥30 s or 12-lead ECG to confirm a diagnosis of clinical AF when detected by screening tools. Although similar observation can be applied to the use of ICM in the same setting, the positive clinical interaction observed in the LOOP trial between high blood pressure values and better clinical outcome in early anticoagulated patients in the ICM arm may prompt the use of an implantable loop recorder (ILR) as a screening tool in selected patients with HTN.

**Figure 4:**
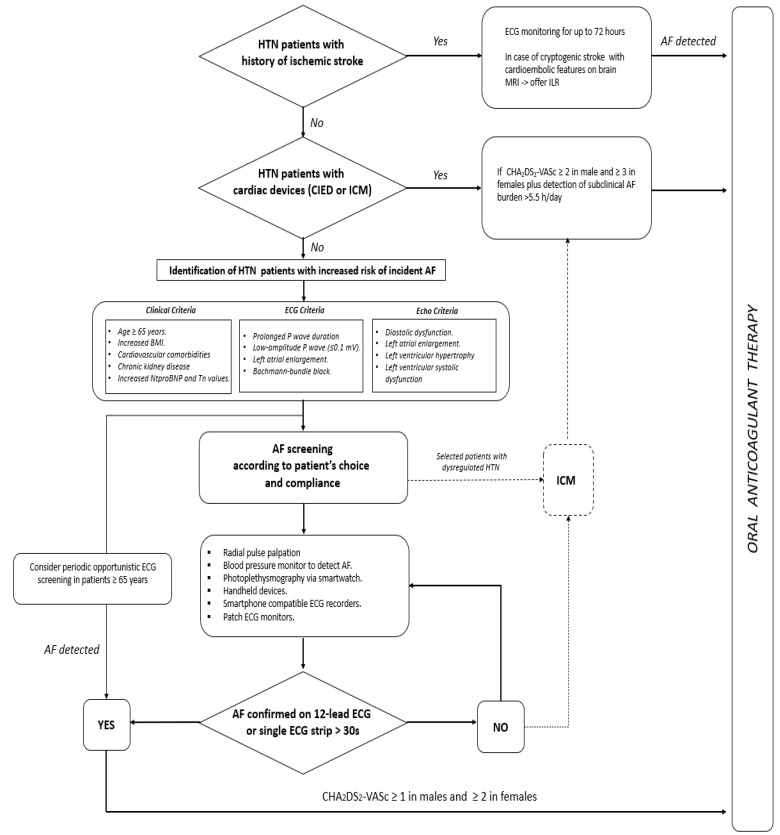
Proposed algorithm for early detection and management of silent and subclinical atrial fibrillation episodes. AF = atrial fibrillation; CIED = cardiac implantable electronic devices; ICM = internal cardiac monitor; ILR = internal loop recorder; HTN = hypertension.

In conclusion, AF detection in its early stage is paramount, and an appropriate therapy might eschew severe complications potentially leading to disability and death in the affected patients. However, it should be ascertained which patients portend a greater risk of AF and thereby who should be screened for this arrhythmia and by which modality. While waiting for sounder results from ongoing clinical trials, Figure 4 provides a proposed algorithm for silent/subclinical AF detection and management in hypertensive patients.

## 6. Conclusions and Future Directions

Through enhanced RAAS and the ensuing pathophysiological mechanisms, HTN represents a well-known substrate for cardiac arrhythmias. Although several trials reported the overall clinical benefit of RAAS inhibitors in reducing AF onset in essential HTN, the role of this class of drugs is greatly reduced when AF diagnosis is already established. Therefore, primary prevention measures are strongly recommended to avoid the potential occurrence of AF in a population already at risk of ischemic and/or haemorrhagic cerebral stroke and, consequently, of disability and death. On the one hand, a patient-tailored, pathophysiology-driven strategy is mandatory in all hypertensive patients, from the administration of RAAS inhibitors in essential HTN to the early detection of secondary HTN causes, namely PA, warranting a specific medical and surgical treatment which has proved to ameliorate the overall outcome in this specific population. 

Finally, although several issues still exist as to the possibility of AF screening in the general population affected by HTN, the early detection of silent/subclinical episodes of AF should be nonetheless carried out while waiting for sounder evidence from ongoing randomised controlled trials in the field.
